# Double heterozygous germline pathogenic variants in patients referred to a tertiary cancer genetics clinic in Singapore

**DOI:** 10.3389/fonc.2026.1903384

**Published:** 2026-08-25

**Authors:** Rachel Su Jen Wong, Pei Yi Ong, Samuel Guan Wei Ow, Robert John Walsh, Gloria Chan, Vanessa Pei Yi Lim, Soo Chin Lee

**Affiliations:** 1 Department of Haematology-Oncology, National University Cancer Institute Singapore, Singapore, Singapore; 2 Cancer Science Institute of Singapore, National University of Singapore, Singapore, Singapore

**Keywords:** Asia, double heterozygosity (DH), double heterozygous pathogenic variants, double mutation, pathogenic variant

## Abstract

**Background:**

The increasing use of multigene panel testing has led to a rise in the identification of double heterozygous (DH) pathogenic variants in cancer patients, although their frequency and clinical relevance remain poorly characterised, particularly in Asian populations.

**Methods:**

We conducted a retrospective review of 5,178 patients referred to a tertiary cancer genetics clinic in Singapore. DH pathogenic variants were defined as the presence of two or more distinct pathogenic or likely pathogenic germline variants identified by multigene panel testing. Clinical, pathological, and family history data were reviewed and analysed using appropriate non-parametric and exact statistical methods.

**Results:**

Among 2,802 index patients with available genetic test results, 593 (21.2%) carried at least one pathogenic/likely pathogenic variant. DH pathogenic variants were identified in 21 individuals (0.75%), of which 9 were patients with breast cancer, 10 with non-breast malignancies, and 2 were cancer-free. The frequency of multiple primary cancers was 26.3% in DH variant carriers, 23.3% in single variant carriers, and 16.5% in those with no pathogenic variants. The median ages of first cancer diagnosis were 47, 44, and 48 years. Several DH combinations involved moderate- or low-penetrance genes, and some clinically unsuspected variants were detected only through broad multigene testing.

**Conclusion:**

DH pathogenic variants represent a rare subgroup that is increasingly detected through multigene panel testing. Their phenotypic expression is variable, and the influence of additional pathogenic variants remains uncertain. Our findings emphasise the need for tailored genetic evaluation and further research to guide evidence-based management for this complex patient population.

## Introduction

In the current era of personalised medicine, the use of germline genetic testing has increased over the years and is expected to continue (1). The availability of targeted drugs approved in some hereditary cancer syndromes in various therapeutic settings has expanded the clinical utility of genetic testing in cancer patients (2). At the same time, advances in next-generation sequencing (NGS) technology have significantly reduced test costs, enabling more mainstream adoption of multigene panel germline testing. Consequently, international guidelines now recommend considering the use of multigene testing in various cancer types and clinical settings (3, 4).

These developments have led to an increase in the detection of germline pathogenic variants in multiple cancer predisposition genes (2, 5). This phenomenon has been described as Multi-locus Inherited Neoplasia Allele Syndrome (MINAS) by Megid et al., to refer to individuals who carry germline pathogenic variants in two or more cancer susceptibility genes (2). For consistency, we will use the term double heterozygous (DH) pathogenic variants throughout this paper to refer to individuals with two or more of such variants.

## Background

The occurrence of DH pathogenic variants is rare, although its true frequency remains uncertain and likely varies depending on the study population and the breadth of genetic testing panels used. The largest published study so far is from a cancer centre in the United States of America (6) that tested 55,803 patients suspected to harbour a hereditary cancer predisposition using a 25-gene hereditary cancer panel and reported DH variants in 106 patients (0.19%).

When focusing specifically on breast cancer patients, several cohort studies have reported varying frequencies of DH variants. A retrospective study of 1,156 breast cancer patients in Brazil identified 14 DH carriers (1.21%) (2). The most commonly affected genes were *BRCA1* (42.9%), *TP53* (35.7%), and *BRCA2* (28.6%). In a German cohort of 8,162 patients with personal and/or family history of breast/ovarian cancer, DH variants in *BRCA1* and/or *BRCA2* were detected in six individuals (0.07%) (7). Meanwhile, an Israeli study of Jewish patients known to carry at least one of the three *BRCA1/2* founder mutations (*BRCA1* 185delAG and 5382insC; *BRCA2* 6174delT) reported a 3.0% frequency of DH variants in *BRCA1* and/or *BRCA2* among 17 out of 567 families (4).

The clinical implications of DH pathogenic variants are currently unclear. It is not well established whether the coexistence of pathogenic variants in two or more high- and/or moderate-penetrance genes lead to additive or synergistic effects that modify cancer risk. Specifically, it is uncertain whether such individuals have an increased likelihood of developing multiple cancers, earlier disease onset, or atypical cancer phenotypes compared with those with a single pathogenic mutation. As a result, evidence-based guidance on the surveillance, risk management, and genetic counselling for these patients is currently limited.

## Methods

We conducted a retrospective review of all patients referred to the Cancer Genetics Clinic at the National University Cancer Institute, Singapore (NCIS), between 1 October 2000 and 6 February 2025. This clinic is within a tertiary cancer institute and receives referrals from public hospitals, private healthcare providers, and self-referred individuals from Singapore and Southeast Asia.

Before 2014, germline genetic testing at NCIS comprised primarily of targeted gene testing using Sanger sequencing and deletion/duplication analysis based on clinical suspicion. From 2014 onwards, NGS-based multigene panels became the standard, incorporating full-gene sequencing and deletion/duplication analysis across a broader range of genes. Over the entire study period, the average number of genes tested per patient was 56 (range 6-216).

DH pathogenic variants were defined as the presence of two or more distinct pathogenic or likely pathogenic germline variants in genes identified by multigene panel testing. A sensitivity analysis was performed restricting DH pathogenic variants to individuals carrying pathogenic or likely pathogenic variants in two established dominant cancer-predisposition genes. Clinical information—including personal cancer history, three-generation family history (including all reported cancer types), cancer type, and histology—was retrieved from clinical records. Statistical analyses were performed using R (version 4.5.2). Analyses of age at first cancer diagnosis and multiple primary malignancies were restricted to cancer-affected individuals, whereas family history analyses included all genetically tested patients. Continuous variables were compared using the Kruskal–Wallis test and categorical variables using Fisher’s exact test. Pairwise comparisons were performed using Wilcoxon rank-sum tests with Bonferroni correction. We examined the association with family history by quantifying the total number of affected first- and second-degree relatives of each individual. A two-sided p < 0.05 was considered statistically significant. The study was approved by the Institutional Ethics Review Board.

## Results

**Figure 1:**
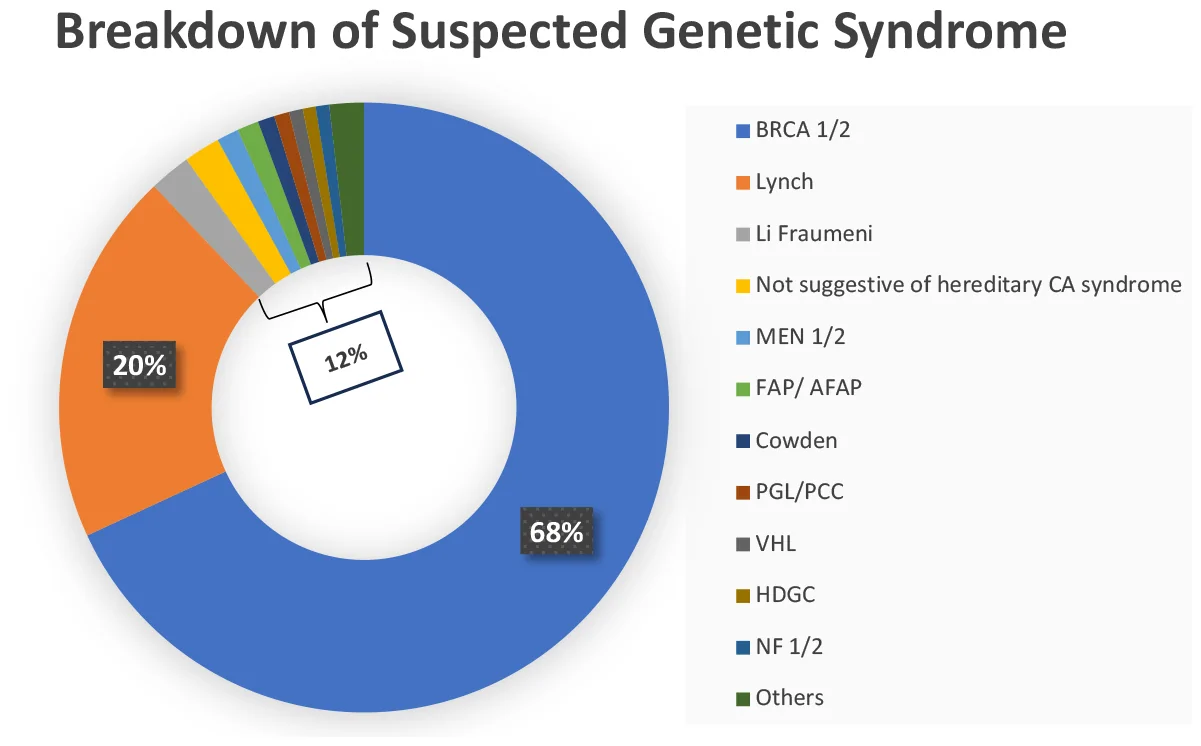
Breakdown of the various suspected syndromes of patients referred to NCIS for genetic testing. MEN 1/2, multiple endocrine neoplasia 1/2; FAP/AFAP, familial adenomatous polyposis/attenuated familial adenomatous polyposis; PGL/PCC, hereditary paraganglioma-pheochromocytoma syndrome; VHL, Von Hippel-Lindau; HDGC, hereditary diffuse gastric cancer; NF1/2, neurofibromatosis types 1/2. Others: hereditary leiomyomatosis and renal cell cancer (HLRCC), Peutz–Jeghers syndrome (PJS), epidermal growth factor receptor (EGFR), colonic oligopolyposis, familial pancreas cancer, hyperplastic polyposis syndrome. Please refer to **Supplementary Table 1** for the detailed percentage breakdown of each suspected syndrome.

A total of 5,178 new patients were seen at the NCIS Cancer Genetics Clinic during the study period. **Figure 1** illustrates the distribution of suspected hereditary syndromes based on their personal and three-generation family cancer histories. A detailed percentage breakdown of each suspected syndrome is presented in **Supplementary Table 1**.

Of the 4,810 index individuals, 2,802 (58.3%) underwent genetic testing and had test results available (the derivation of this cohort is shown in **Supplementary Figure 1**). Among these, 593 individuals (21.2%) were found to carry pathogenic or likely pathogenic variants. The majority of positive cases (96.5%; n=572) harboured a single pathogenic variant. DH pathogenic variants were identified in 21 individuals, accounting for 3.5% among those tested positive for a pathogenic variant and 0.75% of patients who underwent genetic testing. In a sensitivity analysis, restricted only to individuals with pathogenic variants in two established dominant cancer-predisposition genes, 10 of the 21 individuals met the stricter definition, corresponding to a frequency of 0.36%. Given the transition to NGS-based multigene panel testing at NCIS from 2014 onwards, we compared the DH frequency between these two periods. The frequency was 0.50% (1/200) before 2014 and 0.81% (20/2,602) from 2014 onwards. No individual carried pathogenic variants in more than two genes. Hereditary breast and ovarian cancer syndrome (HBOC) was the most common clinically suspected diagnosis (75%) among 2,102 index patients who underwent testing. The rates of DH pathogenic mutations were similar regardless of the clinically suspected syndrome; 0.76% (16/2,102) in those suspected to have HBOC compared with 0.71% (5/700) suspected to have a non-HBOC syndrome.

### Double heterozygous pathogenic variants

DH pathogenic variants were identified in 21 index individuals, including 19 with a personal history of cancer and 2 cancer-free individuals. One cancer-free individual underwent testing due to a personal history of multiple juvenile colonic polyps, whereas the other was an individual of Ashkenazi Jewish descent whose mother had passed on from breast cancer in her thirties and was unavailable for testing. 42.9% (9/21) of patients had primary breast cancer, and 14.3% (3/21) had epithelial ovarian, primary peritoneal, or fallopian tube cancer.

**Figure 2:**
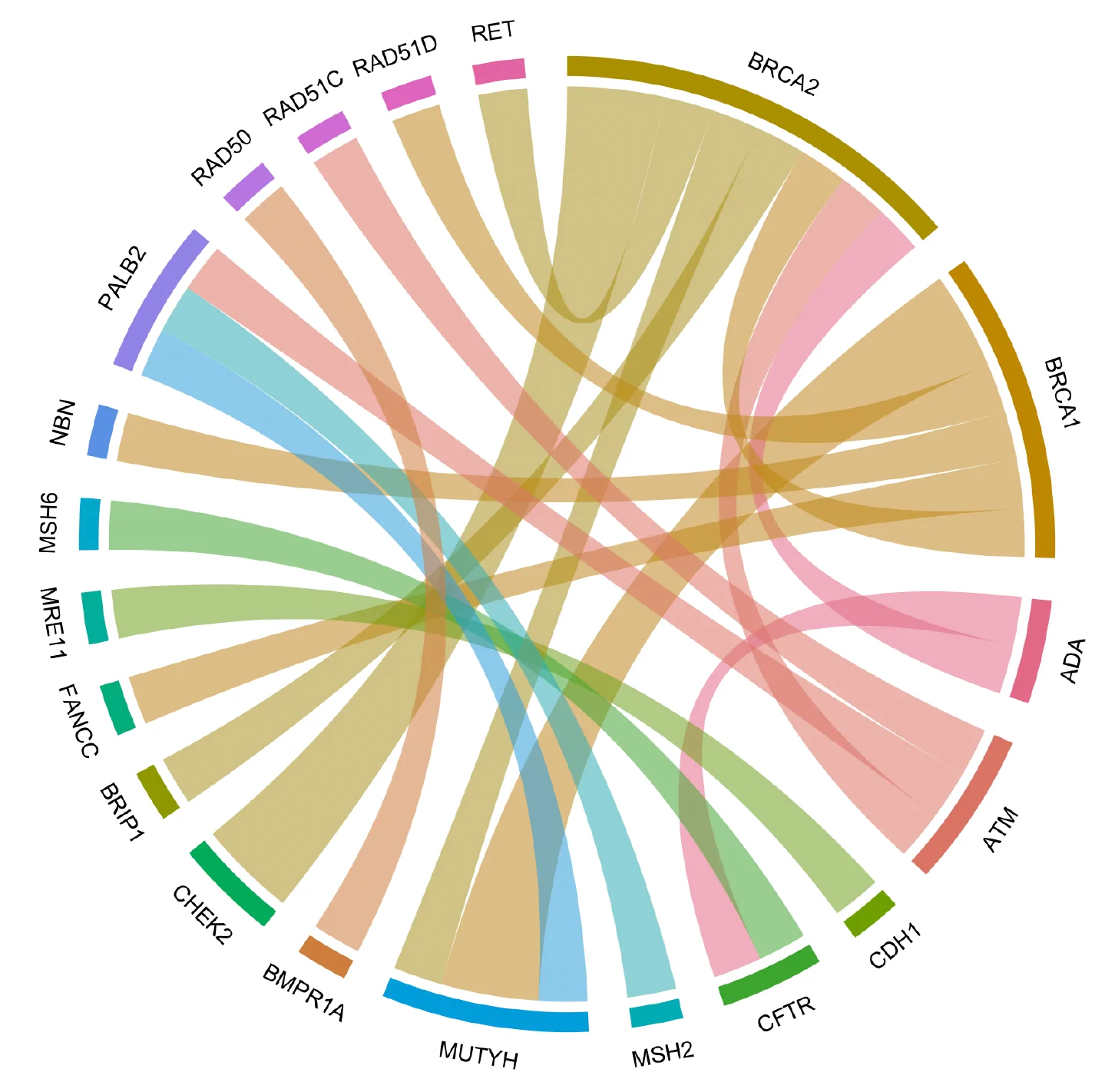
Circos plot (8, 9) illustrating the co-occurring DH pathogenic mutations. Each link represents gene pairs with co-occurring pathogenic variants detected in the same patient. The width of the links reflects frequency. The names of the genes are arranged around the circle.

61.9% (13/21) of DH carriers had at least one *BRCA1/2* pathogenic variant, of which only one patient (4.8%) had DH pathogenic variants in both *BRCA1* and *BRCA2*. 14.3% (3/21) carried a *BRCA1/2* pathogenic variant along with a pathogenic variant in another breast and/or ovarian cancer predisposition gene (*CHEK2*, n=2, *ATM*, n=1). Among the patients with only non-*BRCA1/2* pathogenic variants (n=8), 50% (4/8) carried one pathogenic variant in a known breast and/or ovarian cancer predisposition gene (*PALB2*, n=3, *ATM*, n=1) and another pathogenic variant in a non-breast predisposition gene, whereas 44.4% (4/9) carried DH variants in two non-breast cancer predisposition genes (one each of *ADA + CFTR*, *CDH1 + MRE11, MSH6 + CFTR*, and *BMPR1A + RAD50*). Details of the detected DH variants from these 21 index cases are summarised in **Table 1**, and **Figure 2** illustrates the co-occurring DH pathogenic variants. **Table 2** summarises the clinicopathological characteristics of the cancer patients found to have DH pathogenic variants.

**Table 1:** Detected double heterozygous pathogenic variants from the 21 index cases.

Primary cancer	Gene 1	Gene 2
Breast	*ATM*: c.1321C>T (p.Gln441*) in exon 10*^#^*	*PALB2*: c.3425T>G (p.Leu1142*) in exon 13*^#^*
Breast	*BRCA1*: c.1504_1508del (p.Leu502Alafs*2) in exon 10*^#^*	*MUTYH*: c.733C>T (p.Arg245Cys) in exon 9^⋄^
Breast	*BRCA1*: c.3214del (p.Leu1072*) in exon 10*^#^*	*NBN*: c.1882_1885del (p.Glu628Thrfs*28) in exon 12^‡^
Breast	*BRCA1*: c.4327C>T (p.Arg1443*) in exon 12*^#^*	*RAD51D*: c.270_271dupTA (p.Lys91Ilefs*13) in exon 4*^#^*
Breast	*BRCA2*: C.5722_5723DELCT (P.LEU1908ARGfs*2) in exon 11*^#^*	*MUTYH*: C.394-2A>G (splice acceptor) in intron 10^⋄^
Breast	*BRCA2*: c.475 + 2T>C (splice donor) in intron 5*^#^*	*BRIP1*: c.844dup (p.Thr282Asnfs*6) in exon 7*^#^*
Breast	*BRCA2*: c.5796_5797del (p.His1932Glnfs*12) in exon 11*^#^*	*CHEK2*: c.885_887del (p.Glu295del) in exon 8*^#^*
Breast	*BRCA2*: C.3187_3188INSG (P.GLN1063ARGfs*4) in exon 11*^#^*	*CHEK2*: C.846 + 4_846 + 7DELAGTA (intronic) in intron 7*^#^*
Breast	*BRCA2*: C.5645C>A (P.SER1882*) in exon 11*^#^*	*RET*: C.2410G>A (P.VAL804MET) in exon 14*^#^*
Ovarian	*ADA* c.125_126del (p.Thr42Serfs*24)^†^	*CFTR* c.1210-34TG[12]T[5] (Intronic)^†^
Primary peritoneal carcinoma	*BRCA1*: c.5525delT (p.Val1842Glufs*13) in exon 23*^#^*	*FANCC*: C.1290C>A (P.TYR430*) in exon 13^⋄^
Fallopian tube	*BRCA1*: c.2726dup (p.Asn909Lysfs*6) in exon 10*^#^*	*MUTYH*: c.1435G>T (p.Glu479*) in exon 14^⋄^
Endometrial	*MSH2* c.1076 + 1G>A (Splice donor) in intron 6*^#^*	*PALB2* c.503C>G (p.Ser168*) in exon 4*^#^*
Pancreas	*BRCA2*: c.6832dupA (p. I2278Nfs*15) in exon 10*^#^*	*ATM*: c.4571_4572insAT (p.I1525Lfs*19) in exon 29*^#^*
Ampullary	*ATM* c.2413C>T (p.Arg805*) in exon 16*^#^*	*RAD51C* c.571 + 5G>A (Intronic) in intron 3*^#^*
Ampullary	*BRCA2* c.9154C>T (p.Arg3052Trp) *^#^*	*ADA* c.311C>T (p.Pro104Leu)^†^
Stomach	*CDH1*: c.348_351dup (p.Thr118Glufs*51) in exon 3*^#^*	*MRE11*: c.1726C>T (p.Arg576*) in exon 15^‡^
Colon	*MSH6*: c.3040_3042del (p.Lys1014del) in exon 4*^#^*	*CFTR* c.1210-34TG[13]T[5] (Intronic)^†^
Prostate	*PALB2*: EX4_6del*^#^*	*MUTYH*: c.467G>A (p.W156*) in exon 6^⋄^
Cancer-free	*BMPR1A*: c.127_137del (p.Lys43Trpfs*24) in exon 4*^#^*	*RAD50*: c.1111del (p.Ile371Phefs*8) in exon 8^‡^
Cancer-free	*BRCA1*: 185delAG*^#^*	*BRCA2*: 6174delT*^#^*

*^#^:* Established dominant cancer-predisposition genes.

^⋄^ : Monoallelic variants in recessive cancer-predisposition genes.

^†^ : Carrier-status findings without an established association with cancer risk.

^‡^ : Variants in genes with limited or disputed evidence of cancer predisposition.

**Table 2:** Clinicopathological characteristics of all cancer patients found to have double heterozygous pathogenic mutations.

No.	Primary	Age	Gender	Ethnicity	Histology	Stage	Grade	ER (%)	PR (%)	HER2 (IHC/FISH)	Genes	Multiple cancers
1	Breast	38	Female	Chinese	Ductal carcinoma	II	3	90	80	3+	*ATM PALB2*	
2	Breast	41	Female	Indian	Ductal carcinoma	III	3	90	5	2+/neg	*BRCA1 MUTYH*	
3	Breast	62	Female	Chinese	Lobular carcinoma	II	3	90	80	1+	*BRCA1 NBN*	
4	Breast	40	Female	Chinese	Ductal carcinoma	II	3	0	0	0	*BRCA1 RAD51D*	Stage 1 TNBC, 7 years later
5	Breast	63	Male	Malay	Ductal carcinoma	II	3	90	80	3+	*BRCA2 MUTYH*	
6	Breast	37	Female	Indian	NST	II	3	1-2	1-2	2+/neg	*BRCA2 BRIP1*	
7	Breast	66	Male	Chinese	NST	IV	3	90	15	0	*BRCA2 CHEK2*	
8	Breast	46	Female	Caucasian	Ductal carcinoma	III	3	Pos#	Pos#	Neg#	*BRCA2 CHEK2*	Stage 1 TNBC, 8 years later
9	Breast	49	Female	Malay	Ductal carcinoma	I	3	90	90	Neg#	*BRCA2 RET*	
10	Ovarian carcinoma	29	Female	Chinese	Unknown	I	–	–	–	–	*ADA CFTR*	Breast DCIS, 18 years later
11	Primary peritoneal carcinoma	64	Female	Chinese	Adenocarcinoma	IV	–	–	–	–	*BRCA1 FANCC*	
12	Fallopian tube	58	Female	Malay	High grade serous	#	–	–	–	–	*BRCA1 MUTYH*	
13	Endometrial	46	Female	Malay	Endometrioid adenocarcinoma	IA	–	–	–	–	*MSH2 PALB2*	
14	Pancreas	45	Female	Chinese	Adenocarcinoma	III	–	–	–	–	*BRCA2 ATM*	
15	Ampullary	48	Male	Malay	Adenocarcinoma	I	–	–	–	–	*ATM RAD51C*	
16	Ampullary	59	Female	Chinese	#	#	–	–	–	–	*BRCA2 ADA*	Breast DCIS, 10 years later
17	Stomach	57	Female	Chinese	Adenocarcinoma	#	–	–	–	–	*CDH1 MRE11*	
18	Caecum	41	Female	Chinese	#	II	–	–	–	–	*MSH6 CTFR*	2^nd^: Myxofibrosarcoma, 13 years later 3^rd^: Stage II hormone positive/HER2 negative breast cancer, 17 years later
19	Prostate	59	Male	Chinese	Acinar adenocarcinoma; Gleason (4 + 3)	#	–	–	–	–	*PALB2 MUTYH*	

#: details not available. NST, no special type; TNBC, triple negative breast cancer; DCIS, ductal carcinoma *in situ.*

### Comparing DH carriers with single pathogenic variant carriers and individuals with no pathogenic variants

We compared clinical characteristics of individuals with DH pathogenic variants (n=21), single pathogenic variants (n=572), and no pathogenic variants (n=2,209). The breakdown of cancer patients and healthy individuals in each of the three mutation groups are illustrated in **Supplementary Figure 1**. Analyses were performed in two predefined populations: comparisons of sex, age at first cancer diagnosis, and the presence of multiple primary malignancies were restricted to individuals with a personal history of cancer, whereas analysis of family history was conducted in the full cohort of genetically tested individuals, including those without a cancer diagnosis.

Among cancer-affected patients, the proportion of female patients was similar across mutation groups (78.9% among DH carriers, 81.8% among single pathogenic variant carriers, and 83.6% among those with no pathogenic variants; Fisher’s exact test, p = 0.536). The predominance of female patients across all groups is expected given that most individuals were referred for assessment of suspected HBOC.

Age at first cancer diagnosis differed significantly across mutation groups (Kruskal–Wallis χ² = 44.9, p < 0.001). Median age at first cancer diagnosis was 47 years (IQR 41–58) in patients with DH pathogenic variants, 44 years (IQR 36–53) in those with single pathogenic variants, and 48 years (IQR 40–59) in those with no pathogenic variants. Pairwise comparisons demonstrated a significant difference between single pathogenic variant carriers and those with no pathogenic variants (Bonferroni-adjusted p < 0.001), whereas no significant differences were observed between DH pathogenic variants and either single pathogenic variants (adjusted *p* = 0.33) or patients with no pathogenic variants (adjusted *p* = 1.00). The numerically higher age at first cancer diagnosis observed among DH carriers should be interpreted cautiously given the small number of DH carriers and the lack of a significant pairwise difference. The frequency of multiple primary cancers was 26.3% (5/19) among DH variant carriers, 23.3% (123/527) among single variant carriers, and 16.5% (353/2,136) among those with no pathogenic variants. Pairwise comparison showed no significant difference between DH and single variant carriers (OR 1.17; 95% CI 0.32-3.53; p=0.784).

Breast cancer accounted for 47.4% (9/19) of first primary malignancies among DH carriers, compared with 49.0% (258/527) among individuals with single pathogenic variants and 57.8% (1,234/2,136) among those with no pathogenic variants. The proportion of male breast cancer was higher among DH carriers (22.2%; 2/9) than among single pathogenic variant carriers (2.7%; 7/258) or those with no pathogenic variants (1.5%; 18/1,234); however, this observation is based on a small number of DH cases and should be interpreted cautiously.

Among the entire cohort of individuals with available test results, the presence of a family history of cancer differed significantly across mutation groups (≥1 affected relative in 76.2% of DH carriers, versus 83.7% of single pathogenic variant carriers versus 74.8% of patients with no pathogenic variants; Fisher’s exact test, p < 0.001). When strength of family history was assessed by the total number of affected first- and second-degree relatives, a significant difference was also observed across mutation groups, with single or double pathogenic variant carriers demonstrating a higher median number of affected relatives than patients with no pathogenic variants [median [Q1-Q3]: 2 (1-4) *vs*. 1 (0-3); Kruskal–Wallis χ² = 54.1, p < 0.001]. Given the small number of DH carriers, pairwise comparisons involving this group were considered exploratory due to limited statistical power.

### Notable cases

#### Patient number 14; *MSH2* and *PALB2* co-mutation

Patient #14 (**Table 2**) had DH pathogenic variants in both *MSH2* and *PALB2*. She was an Indonesian woman who was diagnosed with young onset endometrioid endometrial adenocarcinoma at age 46. She underwent a total hysterectomy and bilateral salpingo-oophrectomy and bilateral pelvic lymph node dissection. Histology showed FIGO 1A grade 1 well-differentiated adenocarcinoma, and all 12 lymph nodes removed were negative for malignancy. Her tumour immunohistochemistry staining test showed loss of MSH2 and MSH6 protein expression. Her family pedigree is depicted in **Supplementary Figure 2A**. While the pathogenic *MSH2* variant is concordant with her personal history of endometrial cancer, there is no personal or family history suggestive of a *PALB2* pathogenic variant.

#### Patient number 9; *BRCA2* and *RET* co-mutation

Patient #9 (**Table 2**), an Indonesian woman, was found to carry DH pathogenic variants in *BRCA2* and *RET*. She was diagnosed at age 49 with stage IA, grade 3, hormone receptor-positive, HER2-negative invasive ductal carcinoma of the left breast. Her personal and maternal family history, which included breast and ovarian cancers, were suspicious for HBOC syndrome and concordant with the pathogenic *BRCA2* variant. There was, however, no family history of medullary thyroid cancer or pheochromocytoma in her family. Her family pedigree is shown in **Supplementary Figure 2B**.

Given the unexpected *RET* pathogenic variant and discordant family history, she was offered confirmatory testing using repeat *RET* mutation analysis in a different laboratory, which verified the presence of the *RET* variant. Cascade testing was offered to her daughters: one daughter tested positive for both the *BRCA2* and *RET* pathogenic variants, whereas another tested negative for both variants. The patient was subsequently initiated on surveillance for medullary thyroid carcinoma, pheochromocytoma, and hyperparathyroidism as recommended for *RET* mutation carriers.

## Discussion

To our knowledge, this is the largest reported series of patients with DH pathogenic variants reported on multigene panel testing from Asia. Among similar reported series worldwide, only two were larger studies with a sample size approximating 100 patients (Weitzel et al. from USA, n=106 (6); Rebbeck et al. from an international consortium, n=93 (10). Of the remaining published studies, one reported 22 DH carriers, whereas the others reported cohorts comprising fewer than 20 patients. Our series of 21 unrelated DH carriers thus ranks among the largest reported from a single institution to date and the largest in Asia.

Across diverse populations and testing strategies (**Table 3**), the frequency of DH pathogenic variants remains consistently low, ranging from approximately 0.07% to 3% (2, 6, 7, 10–14).

**Table 3:** Summary of the available studies reporting the frequency of double heterozygous pathogenic variants.

Region (reference)	Population tested	Sample size	Genes tested (*n*)	DH carriers (*n)*	DH frequency
DH not restricted to *BRCA1/2*					
Singapore (current study)	Index patients with any suspected hereditary syndrome	2,802	56 (average)	21	0.75%
	▪ HBOC syndrome	2,102		16	0.76%
	▪ Non-HBOC syndrome	700		5	0.71%
Brazil (2)	Breast cancer patients	1,156	At least 12	14	1.21%
USA (6)	Patients with suspected hereditary cancer predisposition	55,803	25	106	0.19%
Russia, Belarus, Poland (12)	Breast cancer patients of Slavic ancestry	5,391	5^*^	17	0.32%
Hong Kong (13)	High-risk breast cancer patients	3,649	30 or 93	9	0.25%
Turkey (15)	Patients with suspected hereditary cancer syndromes	655	60 or 149	14	2.14%
*BRCA1/2*-only DH					
Germany (7)	Breast and/or ovarian cancer patients	8,162	2 (*BRCA 1/2*)	6	0.07%
International consortium (10)	Female *BRCA1/2* mutation carriers	32,295	2	93	0.29%
Israel (11)	Breast cancer patients of Ashkenazi Jewish ancestry	1,191 (from 567 families)	3 *BRCA* founder mutations^#^	22 (from 17 families)	3.0% ^#^
Korea (14)	High-risk breast cancer patients	4,215	2 (*BRCA 1/2*)	7	0.17%

*: BRCA1, CHEK2, NBN/NBS1, ATM, and BLM.

*♢*: DH frequency reported at the family level as DH status assumed to be shared within families.

#: 185delAG and 5382insC on *BRCA1* and 6174delT on *BRCA2.*

Among DH carriers restricted to *BRCA1/2*, reported frequencies vary by population and are highest in founder-enriched cohorts. Notably, in an Ashkenazi Jewish cohort with high prevalence of three *BRCA* founder mutations, Lavie et al. (11) identified 17 out of 567 families to carry DH in *BRCA1/2* (3.0%). In contrast, Heidemann et al. (7) detected 6 *BRCA1/2* DH carriers (0.07%) among 8,162 German patients with breast and/or ovarian cancer histories, whereas an international consortium (10) reported 93 women with *BRCA1/2* DH variants among 32,295 *BRCA1/2* carriers (0.29%). Of note, the frequency of *BRCA1/2* DH variants is very low in our study (1/2802; 0.04%), which comprised largely Southeast Asian patients with no major *BRCA* founder mutations.

DH carriers harbouring pathogenic variants not restricted to *BRCA1/2*—defined as individuals carrying at least one pathogenic variant in a non-*BRCA* cancer predisposition gene, with or without a concurrent *BRCA1/2* mutation—have been less frequently reported. Published data include a Brazilian cohort (2) of 1,156 breast cancer patients, in which 14 such DH carriers were identified (1.21%); a large U.S. cancer centre reporting 106 DH carriers among 55,803 individuals (0.19%) undergoing testing for suspected hereditary predisposition (6); and a smaller study from Turkey (2), which identified 14 DH carriers amongst 655 patients (2.14%) with suspected hereditary cancer syndromes. In a cohort of 5,391 breast cancer patients from Russia, Belarus, and Poland with known Slavic founder alleles, Sokolenko et al. (12) identified 17 DH carriers (0.32%). In comparison, the frequency of DH carriers in our study is 0.75%, and this was reduced to 0.36% when restricted to individuals with pathogenic variants in two established dominant cancer-predisposing genes.

Within Asia, published data on germline DH pathogenic variants remain scarce. Three studies from Hong Kong (13), Korea (14), and Turkey (15) described 9, 7, and 14 carriers, respectively, corresponding to frequencies of 0.25%, 0.17%, and 2.14% among tested patients. Both the Hong Kong and Korean studies focused on highly selected, high-risk breast cancer populations, although the Hong Kong study employed a relatively broad multigene panel.

In contrast, both the Turkish study and ours assessed more diverse patient populations using broad multigene panel testing, resulting in a wider spectrum of genotypes. Accordingly, the frequency of DH pathogenic variant was higher in these cohorts, at 2.14% and 0.75%, respectively. When we compared the DH frequencies between individuals tested before and from 2014 onwards in our cohort, the observed frequency was numerically higher following the introduction of larger NGS-based multigene panels (0.81% [20/2,602] *vs*. 0.50% [1/200]). However, this comparison should be interpreted with caution, as the size and composition of the genetic testing panels evolved over the study period. Consequently, the reported DH frequency should be interpreted in the context of the evolving genetic testing strategy used in our cohort.

Among breast cancer patients with DH variants, 33.3% of the Turkish cohort and 44.4% of our cohort carried pathogenic variants in two moderate-to-high penetrance breast cancer genes, compared with 77.8% in the Hong Kong study and 100% in the Korean study, likely reflecting the differences in breadth of gene panels used in the different studies. The remaining patients in our cohort harboured pathogenic variant combinations involving moderate-penetrance breast cancer predisposition genes or non-breast cancer predisposition genes, several of which have clear implications for cancer surveillance and risk management.

The impact of a broader testing strategy is further illustrated by analysis of our HBOC cohort. Had *BRCA1/2*-only gene analysis been performed, 11.1% (1/9) of breast cancer patients and 33.3% (1/3) of patients with ovarian, fallopian tube, or primary peritoneal carcinoma would not have been identified as carrying any pathogenic variant. Consequently, none of these individuals would have been recognised as DH pathogenic variant carriers, underscoring how broader multigene testing and inclusive referral criteria can substantially influence both the detection rate and clinical characterisation.

The clinical significance of harbouring a second pathogenic variant remains uncertain; however, our findings offer some insight into potential phenotypic effects (**Table 4**). The proportion of bilateral breast cancers among DH carriers in our cohort was 2/9 (22.2%), compared with 4/9 (44.4%) in the Hong Kong study and 1/7 (14.3%) in the Korean study. In our cohort, both affected patients developed contralateral breast cancers more than 5 years after their initial diagnosis, with both second primary cancers presenting as early-stage TNBC. Although each patient carried a pathogenic *BRCA1* or *BRCA2* mutation—sufficient to explain the observed cancer phenotype—they also harboured pathogenic variants in moderate-penetrance cancer predisposition genes (*CHEK2* and *RAD51D*, respectively).

**Table 4:** Summary of four Asian case series (including our study) reporting double heterozygous pathogenic variants.

	Singapore	Hong Kong (13)	Korea (14)	Turkey (15)
Patient population	Patients suspected of hereditary syndrome	High-risk breast cancer patients	High-risk breast cancer patients	Patients suspected of hereditary syndrome
Number of patients tested	2,802	3,649	4,215	655
Number of genes tested	Average 56 genes (range 6-216)	30 gene panel or 93 gene panel	2 genes (BRCA 1/2)	60 or 149 gene panel
Double heterozygous (DH) pathogenic variant rate	21/2,802 (0.75%)	9/3,649 (0.25%)	7/4,215 (0.17%)	14/655 (2.14%)
Breast cancer patients with DH	9 patients	9 patients	7 patients	6 patients
▪ Bilateral breast cancer rate	2 (22.2%)	4 (44.4%)	1 (14.3%)	#
▪ Median age at first cancer diagnosis (range)	46 (37-66)	36 (30-69)	35 (26-42)	#
▪ Triple-negative breast cancer	1 (11.1%)	5 (55.6%)	5 (71.4%)	#
▪ Grade 3 tumours	9 (100%)	Unknown	4 (57.1%)	#
▪ Both DH genes ≥ moderate risk penetrance for breast cancer	4 (44.4%)	7 (77.8%)	7 (100%)	2 (33.3%)

#: details not available.

These observations raise the possibility that secondary mutations could influence penetrance or modify cancer risk. However, current evidence is inconclusive and does not support a consistent genotype–phenotype correlation in DH carriers. While Heidemann et al. (7) suggested that female DH carriers may have a higher probability of developing cancer, earlier onset, and more severe disease compared with carriers of a single *BRCA* mutation, similar associations were not observed in studies by Lavie et al. (11) or Megid et al. (2). In our cohort, we observed a higher proportion of male breast cancer among DH carriers compared with individuals carrying single pathogenic mutations or a negative test, although this observation was based on small numbers and should be interpreted with caution. Age at first cancer diagnosis differed significantly across mutation groups overall, but this difference was driven by single pathogenic variant carriers compared with those with no pathogenic variants.

Taken together, it remains equally plausible that the occurrence of second primary cancers in DH carriers arose primarily due to high-penetrance *BRCA* mutations rather than a synergistic effect of multiple pathogenic variants. Larger datasets with longitudinal follow-up will be crucial to clarifying whether additional pathogenic variants contribute meaningfully to cancer risk or clinical behaviour.

Most published case reports and studies on DH pathogenic variants have focused primarily on patients with breast cancer. In contrast, our study was not restricted to a single cancer type, although the majority of patients tested were breast cancer patients (53%). The overall frequency of multiple primary cancers in our cohort was 24.0% among cancer patients with a single pathogenic mutation and 26.3% among cancer patients with DH pathogenic variants. Pairwise comparison showed no significant differences between these groups. These findings should be interpreted with caution, as follow-up duration and other clinical factors may influence the occurrence of multiple primary cancers. As these analyses were not adjusted for follow-up, the findings should be regarded as exploratory. Notably, 10 DH carriers in our series presented with non-breast primary cancers, including malignancies of the stomach, colon, prostate, pancreas, or ampulla, and female reproductive tract. Among these, three patients (30%) developed more than one primary malignancy. With the exception of one patient carrying *BRCA2* and *ADA* co-mutations—who was initially diagnosed with ampullary cancer and subsequently developed DCIS of the breast a decade later—the other two patients (patients 10 and 18) developed second cancers that are not currently recognised to have established associations with their respective underlying germline mutations. These observations raise important questions about the potential interactions between co-occurring mutations, although implications for genetic counselling and surveillance recommendations remain uncertain and challenging to define.

Among our cohort, two patients were identified with DH pathogenic variants involving distinct cancer predisposition pathways, one of which was not clinically suspected based on personal and/or family history: *MSH2/PALB2* and *BRCA2/RET*. Both cases exemplify the complexity of genotype–phenotype correlations and highlight the influence of incomplete or tissue-specific penetrance. The *MSH2/PALB2* carrier developed early-onset endometrial carcinoma with tumour loss of MSH2/MSH6 expression, consistent with a Lynch syndrome phenotype, whereas the concurrent pathogenic *PALB2* variant remained clinically silent up to the age of 47. This might be explained by the fact that *PALB2* mutations are known to have incomplete penetrance (16), conferring an estimated lifetime breast cancer risk of approximately 33%-58% in female carriers (17). Similarly, the *BRCA2/RET* carrier presented with hormone receptor-positive, HER2-negative breast cancer typical of *BRCA2*-related hereditary breast and ovarian cancer, without clinical evidence of RET-associated disease as of age 49. Her *RET*: c.2410G>A (p.Val804Met) variant, which has been reported to segregate with medullary thyroid cancer in several families with a penetrance of 30%-40% (16), likely represents a case of reduced expressivity in this context. A clear genotype–phenotype correlation in such multigene carriers therefore remains uncertain, particularly as broader multigene testing may identify pathogenic variants that are not currently associated with the presenting cancer phenotype according to established clinical guidelines (e.g., NCCN (18)). Counselling cancer-free individuals who harbour these variants is particularly challenging in the absence of an established tumour phenotype. Longitudinal follow-up remains essential to refine risk prediction and guide appropriate surveillance.

Lastly, we identified several DH pathogenic variant combinations that involved moderate- or low-penetrance genes such as heterozygous *MUTYH* or *MRE11* or *CFTR*. These findings were unexpected based on the patients’ personal and/or family history, and current management guidelines for these genes are either limited or evolving. This highlights an important trade-off inherent in broader multigene panel testing; whereas such panels increase the detection of clinically relevant hereditary cancer risks that may not be predicted by phenotype alone, they also increase the likelihood of uncovering variants in lower-penetrance genes for which cancer risks and recommended management remain uncertain. As multigene testing becomes more widely used, cautious interpretation of these findings and thoughtful, context-specific genetic counselling will be essential to supporting appropriate clinical decision-making.

## Conclusion

Although uncommon, cases of DH pathogenic variants are being increasingly identified through multigene panel testing. In our cohort, DH carriers demonstrated a heterogeneous cancer spectrum, including both breast and non-breast malignancies, with a considerable proportion developing multiple primary tumours. The presence of high- and moderate-penetrance mutations complicates risk stratification, surveillance strategies, and genetic counselling—particularly when genotype–phenotype correlations are unclear or when counselling cancer-free individuals. Further studies are needed to better define cancer risks and clinical outcomes associated with DH pathogenic variants to inform clinical practice and counselling strategies and to optimise care for this unique subgroup.

## Data Availability

All relevant data is contained within the article: The original contributions presented in the study are included in the article/**Supplementary Material**, further inquiries can be directed to the corresponding author.
