# Supplementary material for: Double heterozygous germline pathogenic variants in patients referred to a tertiary cancer genetics clinic in Singapore
Source: Front Oncol. 2026 Aug 25;16:1903384. doi: 10.3389/fonc.2026.1903384 (PMC13560775; doi:10.3389/fonc.2026.1903384)
Supplement: Supplementary file 1 [file Supplementaryfile1.pdf]

Supplementary Tables and Figures

Suspected Syndromes of Patients	Percentage (%)
BRCA 1/2	68.1
Lynch	19.8
Li Fraumeni	2.2
Not suggestive of hereditary cancer syndrome	1.9
MEN 1/2	1.2
FAP/ AFAP	1.1
Cowden	0.9
PGL/PCC	0.8
VHL	0.7
HDGC	0.7
NF1/2	0.7
Others	1.8

Supplementary Table 1: Detailed percentage breakdown of each suspected syndromes of patients referred to NCIS for genetic testing

Abbreviations: MEN 1/2 – multiple endocrine neoplasia 1/2; FAP/AFAP – familial adenomatous polyposis/ attenuated familial adenomatous polyposis; PGL/PCC - hereditary paraganglioma-pheochromocytoma syndrome; VHL – Von Hippel-Lindau; HDGC – hereditary diffuse gastric cancer; NF1/2 – neurofibromatosis types 1/2
Others: Hereditary Leiomyomatosis and Renal Cell Cancer (HLRCC), Peutz-Jeghers syndrome (PJS), Epidermal Growth Factor Receptor (EGFR), colonic oligopolyposis, familial pancreas cancer, hyperplastic polyposis syndrome

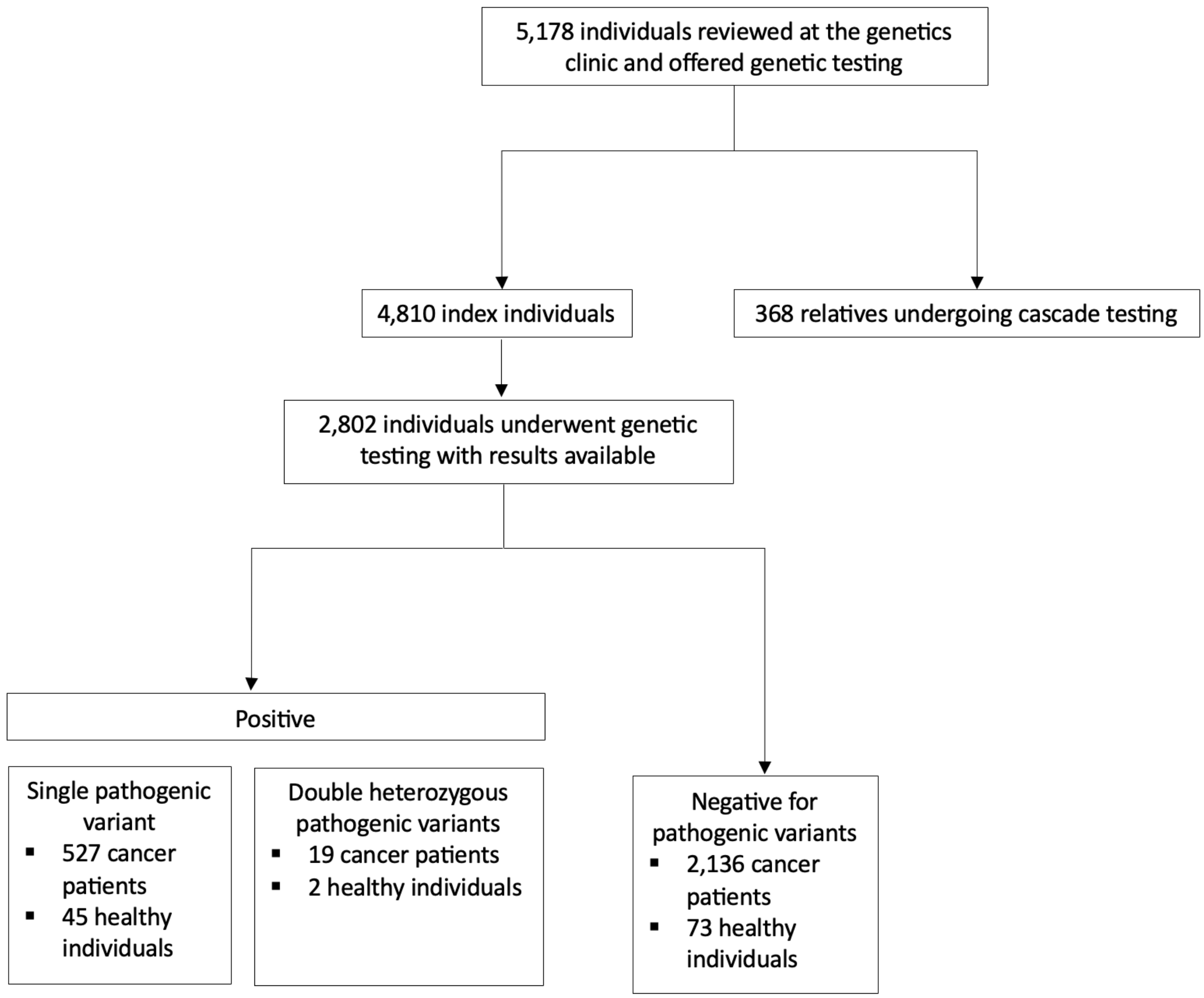

Supplementary Figure 1: Flow diagram of patients referred to the genetics clinic

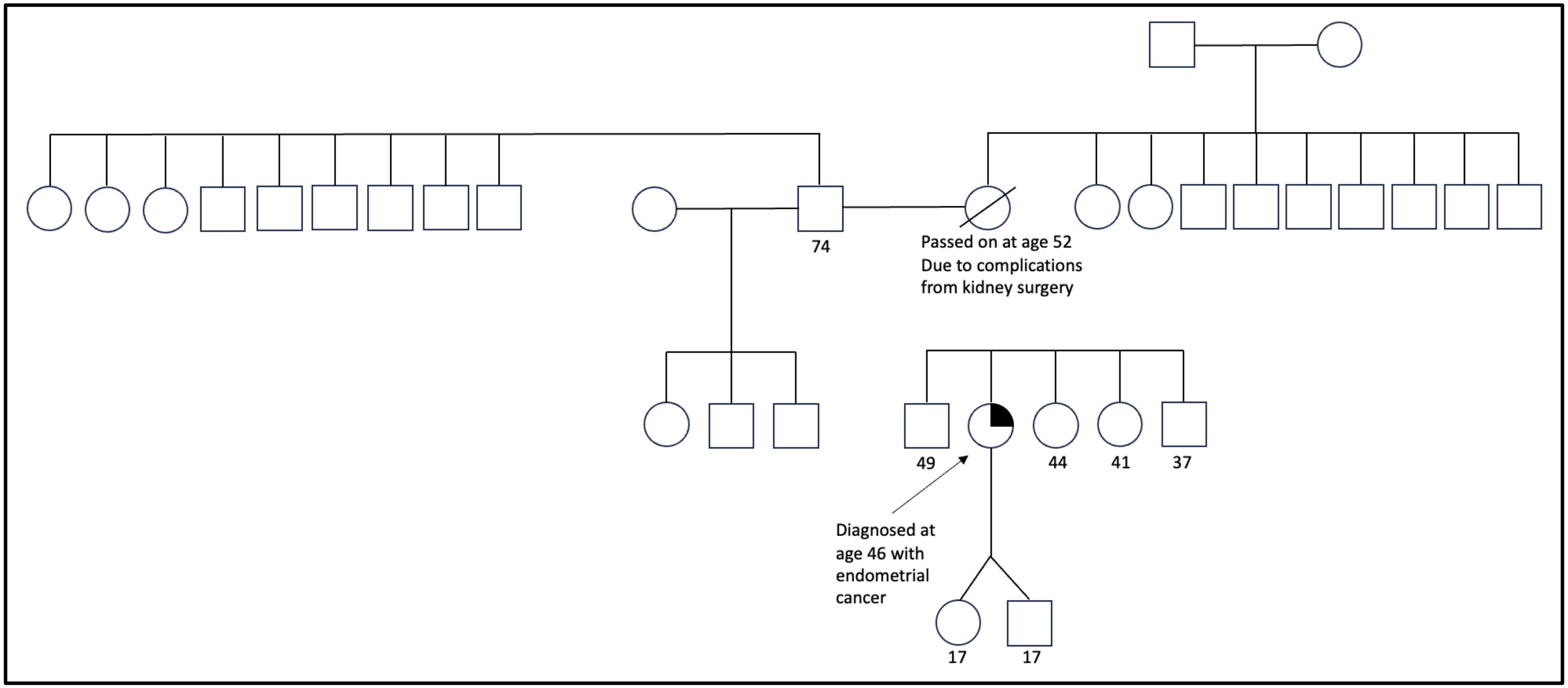

Supplementary Figure 2A: Genogram of patient found to have *MSH2* and *PALB2* co-mutation

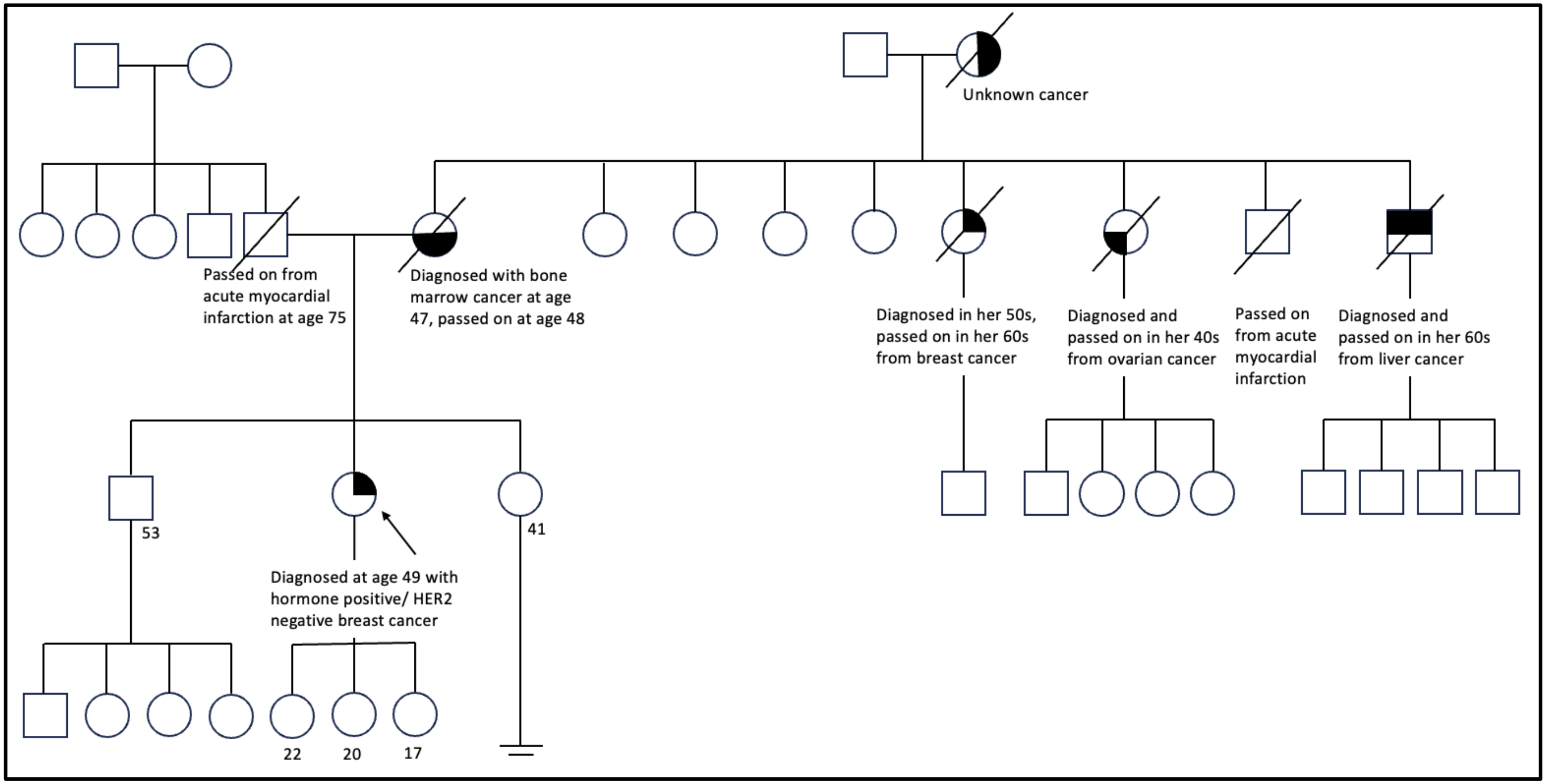

Supplementary Figure 2B: Genogram of patient found to have *BRCA2* and *RET* co-mutation
